# Large CO_2_ reduction and enhanced thermal performance of agro-forestry, construction and demolition waste based fly ash bricks for sustainable construction

**DOI:** 10.1038/s41598-024-59012-8

**Published:** 2024-04-10

**Authors:** Siddharth Singh, Soumitra Maiti, Ravindra Singh Bisht, Soraj Kumar Panigrahi, Sameer Yadav

**Affiliations:** 1 Construction, Automation and Robotics Group, CSIR- Central Building Research Institute, Roorkee, 247667 India; 2 Building Materials and Environmental Sustainability Group, CSIR- Central Building Research Institute, Roorkee, 247667 India

**Keywords:** Agro-forestry waste, Construction & demolition (C&D) waste, Fly ash bricks, Life cycle analysis, Embodied energy, Thermal properties, Environmental sciences, Engineering

## Abstract

The exhaust gases in production of burnt clay bricks is responsible for greenhouse gases (GHGs) emission which increase the carbon footprint in the ecosystem. Here, we report carbon emission and thermal performance based evaluation of 8 ft. × 9 ft. × 8 ft. building. The bricks used in building construction are manufactured from fly ash, agro-forestry wastes, construction & demolition wastes (C&D), ground granulated blast furnace slag (GGBFS) using NaOH as activator in order to provide compressive strength in the range of 3–6 MPa with ambient curing at 30 °C for 28 days. Life cycle analysis (LCA) reveals the total CO_2_ emission for fly ash and burnt clay bricks estimated to be 43.28 gCO_2_ and 290 gCO_2_ per brick, respectively. Considering the current scenario, by replacing 1–2% of brunt clay bricks with agro-forestry waste, C&D waste based fly ash bricks can potentially reduce 0.5–1.5 million tons of CO_2_ emission annually. The embodied energy calculation shows fly ash based bricks consumes 10–15 times less energy as compared to burnt clay bricks. Thermal paremeters viz., U-value (0.5–1.2 W/m^2^K), thermal conductivity (0.4–0.5 W/mK) show adequate insulation of agro-forestry waste based fly ash bricks highlighting its importance of thermal comfort, CO_2_ reduction along with sustainable and eco-friendly construction practices.

## Introduction

Production of 1-burnt clay brick and 1-ton of cement results in approximately 0.41 kg and 0.85 ton of CO_2_ emission^1–3^. Utilisation of farm soil in burnt clay bricks reduces the fertility of agricultural soil, mining of natural aggregates, results in degradation and polluting river banks, poor air and agricultural land quality resulting in ecological imbalance. On an average, the fluoride concentration in soil is about 500 ppm which is released in the atmosphere from the brick kilns when fired at 1000 °C along with other harmful gases viz., CO_2_, SO_2_ and NO_x_ posing threat to human health with acute respiratory problems^4^. The global production of clay bricks will reach to 1.5 trillion contributing to 20$\%$ of global black carbon emission. As per literature, India’s current requirement of burnt clay bricks is approximately 200–250 billion bricks will soon rise to 700 billion bricks in infrastructure sector. According to world bank, the annual production of clay bricks in India is around 200 billion requiring 62 million tons of coal for its production. This will results in 41.6 million tons of CO_2_ emission^5^. Most of the brick manufacturing kilns are in northern part of India situated on the Indo-Gangetic plain comprising of fertile alluvial soil expanding over 700,000 km^2^ region. Approximately 60,000–70,000 brick kiln found in this region are fixed chimney bull’s trench kiln (FCBTK) which are old and traditional type kiln resulting in increased particulate matter and black carbon emission^6–8^.

Apart from the traditional building materials, waste generated from coal based power plants in the form of ash is also responsible for landfill and air pollution, and its proper utilisation is a huge challenge for energy and construction sector. According to a latest report by Ministry of Power, Govt. of India, the total fly ash generated form thermal power plants in India is approximately 270.82 million tons and out of which 259.86 million tons is utilised by various sectors with a utilisation of 95.95 percent^9^. The remaining fly ash may still cause serious land fill issues and land degradation problem if not properly utilised or disposed. The waste generated from construction and demolition activities in construction sector leads to degraded air quality with large amount of particulate matter (PM) and fine aerosols suspended in the atmosphere leading to respiratory problems in the vicinity areas. Between 2020 and 2021, major Indian cities have generated around 150 million tons of C&D waste^10^. Ground granulated blast furnace slag (GGBFS) which is a waste generated from iron and steel industry has shown a significant concern regarding its utilisation and proper disposal. According to the literature total amount of GGBFS production will reach around 270 million tonnes between 2020 and 2025 globally. In India, as per the study of Indian bureau of Mines, the annual GGBFS production is approximately be greater than 17 million tons with its major utilisation in slag based cements^11–13^.

Similarly, the wastes generated on the farm fields after crop harvesting is also a major issue in agricultural sector. In India, over 500 million tons of agricultural residue is generated annually^14,15^. The surplus agricultural residue which mostly accounts for rice and wheat, out of which 60% is burnt mostly at the farm fields^16,17^. The burning of these crop residues results in severe air pollution and poor air quality index in the nearby regions^18^. Similarly burning of forestry leaves also results in air pollution, fire hazards and black carbon emission.

Research on alkali-activated fly ash (AA-FA) with incorporation of various wastes as sustainable building materials has been done to address the waste management issues. Detailed insight on mechanical properties based on co-existence of sodium aluminosilicate gel (N–A–S–H) and calcium aluminosilicate hydrate (C–A–S–H) in presence of sodium silicate and NaOH solution of different molarities, with mechanical strength of 6–16 MPa have been reported by various researchers. Microstructural and mechanical properties of burnt clay bricks using C&D waste over the range of 0–100% have also been studied with compressive strength ranging from 5 to 15 MPa and thermal conductivity of 0.07–0.12 W/m K. Agricultural and industrial wastes-based bricks have been investigated in alkali activated fly ash by various researchers^19–23^. Various supplementary cementitious materials (SCMs) viz., fly ash (FA), GGBFS, silica fume (SF) utilisation in alkali activated concrete have shown to reduce GHGs emission and landfill problems. It is reported that addition of these SCMs in cement can reduced 13–22% of CO_2_ emissions^24,25^. However, the incorporation of treated/untreated agro-waste straw fibres, dried forestry leaves along with C&D wastes in construction materials has been scarcely reported. The effect of agro-forestry content viz., rice straw, dried forestry leaves with C&D waste and GGBFS addition on mechanical, thermal, acoustical and fire related properties of alkali activated fly ash bricks have been done in detail in the authors’ previous study^26^. The study has shown promising results of agro-forestry waste addition along with slag and C&D waste in fly ash bricks with 5 M alkali solution with compressive strength of 8–15 MPa, acoustic isolation of 35–50 dB and thermal conductivity of 0.4–0.5 W/m K.

The manufacturing of construction materials viz., bricks, cement and concrete etc., result in greenhouse gases (GHGs) emission causing a major disturbance in the ecosystem with rising global temperature leading to climate change. To evaluate the CO_2_ emission at various stages of a construction material production, life cycle assessment (LCA) is most widely used approach for determining the overall environmental impact^27,28^. LCA is a popular and rigorous method for assessing the overall environmental impact of building materials over its complete life cycle. The CO_2_ emission at various stages viz., extraction of materials, chemicals (NaOH) production resulting in 1.915 ton-CO_2_ emission per ton production, transportation of materials, fuel requirement, brick manufacturing, firing/treatment and storage/distribution steps must be considered while calculating the total CO_2_ emissions and over all environmental assessment^29^.

Apart from the CO_2_ emissions caused by building material production, providing thermal comfort to a building with external aids is also responsible for greenhouse gases (GHGs) emission. Improvement in energy efficiency of the building for optimum energy consumption in context with thermal comfort plays an important role in reducing the GHGs emission. In a study on dynamic thermal performance of conventional and alternative building wall envelopes provides a good insight regarding steady and dynamic state analysis of various building materials viz., porous autoclaved aerated, fly ash gypsum based bricks, conventional burnt clay bricks etc., and their respective building envelopes^30^. Authors’ previous study on dynamic and steady state thermal analysis on cement fibreboard and bamboo based buildings has elaborated parameters viz., U-value, thermal resistance, decrement factor, time lag and thermal admittance properties in detail^31^.

The present study explores various agricultural and industrial waste viz., fly ash, GGBFS, rice straw, forestry leaves and C&D waste based bricks in building construction. Investigations regarding life cycle assessment involving CO_2_ emission, embodied energy and thermal performance behaviour have been done in detail to provide a holistic framework for further research in the field of sustainable building products. In the present study, the authors report to minimise the CO_2_ emission by utilisation of fly ash, C&D waste and agro-forestry waste in construction of 8 ft. × 9 ft. × 8 ft. masonry building and compared with burnt clay bricks building. A study on CO_2_ emissions caused by agro-forestry, C&D waste based fly ash bricks and its comparison with burnt clay bricks, embodied energy calculation along with thermal (building envelope) analysis are reported and discussed in detail. The present work may be considered as the continuation of authors’ previous work done on properties evaluation of agro-forestry waste and C&D waste based fly ash bricks^26^.

The main objectives in the current study may be defined as:

- Estimation of carbon emission, embodied energy of fly ash and burnt clay bricks.
- Estimation of steady state parameters for agro-forestry waste, C&D waste based fly ash bricks and burnt clay bricks.
- Evaluation of dynamic responses viz., time lag, decrement factor, thermal damping and thermal admittance.

## Materials and methods

### Raw materials

Alkali-activated fly ash (obtained from thermal power plant, NTPC Dadri, Uttar Pradesh, India) bricks incorporating a mixture of locally available agro-waste (rice straw) from farm fields, dried bamboo leaves, syzygium cumini, mangifera indica as forestry waste mixed in equal proportion by weight percent in crushed form, GGBFS from Tata Steel Sahibabad, Uttar Pradesh, India, were used as raw materials. C&D waste from local demolition site consisting of red bricks, mortar, and concrete components as the constituents of fly ash bricks has been investigated in the present study. The fly ash collected from the thermal power plants is usually obtained from burning bituminous and anthracite coals which have high ash content (30–40%) and low calcium content (1–5%), classified as class ‘F’ fly ash characterised by x-ray fluorescence spectroscopy (XRF) method (Bruker S4 Poinner). 3 M sodium hydroxide (NaOH > 98%, Thomas Baker) solution with water to binder ratio of 0.4–0.5 was employed for brick casting.

The agro-forestry wastes were dried under ambient sunlight to remove excess moisture (up to 5%) that may cause any fungal or biodegradation of the stockpile and chopped to desired 5 mm of length of 3–5 mm thickness. The chemical compositions of fly ash which includes crystalline phases of quartz (SiO_2_), mullite (Al_2_O_3_), and hematite (Fe_2_O_3_) as major components and CaO, K_2_O, TiO_2_, MgO and MnO as minor components. Chemical composition of fly ash and GGBFS with physical properties viz., fineness, particle size have been mentioned in detail in Table 1.

**Table 1:** Chemical constituents of fly ash and GGBFS^26,32^.

Chemical constituents	GGBFS (content %)	Fly ash (content %)
SiO_2_	28.3	58.56
Al_2_O_3_	18.0	28.39
Fe_2_O_3_	0.3	4.82
SiO_2_ + Al_2_O_3_ + Fe_2_O_3_	46.6	91.77
SO_3_	3.4	0.54
Na_2_O	4.0	0.09
K_2_O	1.93	0.89
TiO_2_	1.81	2.63
CaO	29.2	1.98
MgO	8.4	0.9
MnO	0.8	0.12
Loss on ignition	6.7	1.08
Blaine fineness (m^2^/kg)	339	394
Average particle size (µm)	25.1	25.6

### Brick manufacturing

**Figure 1:**
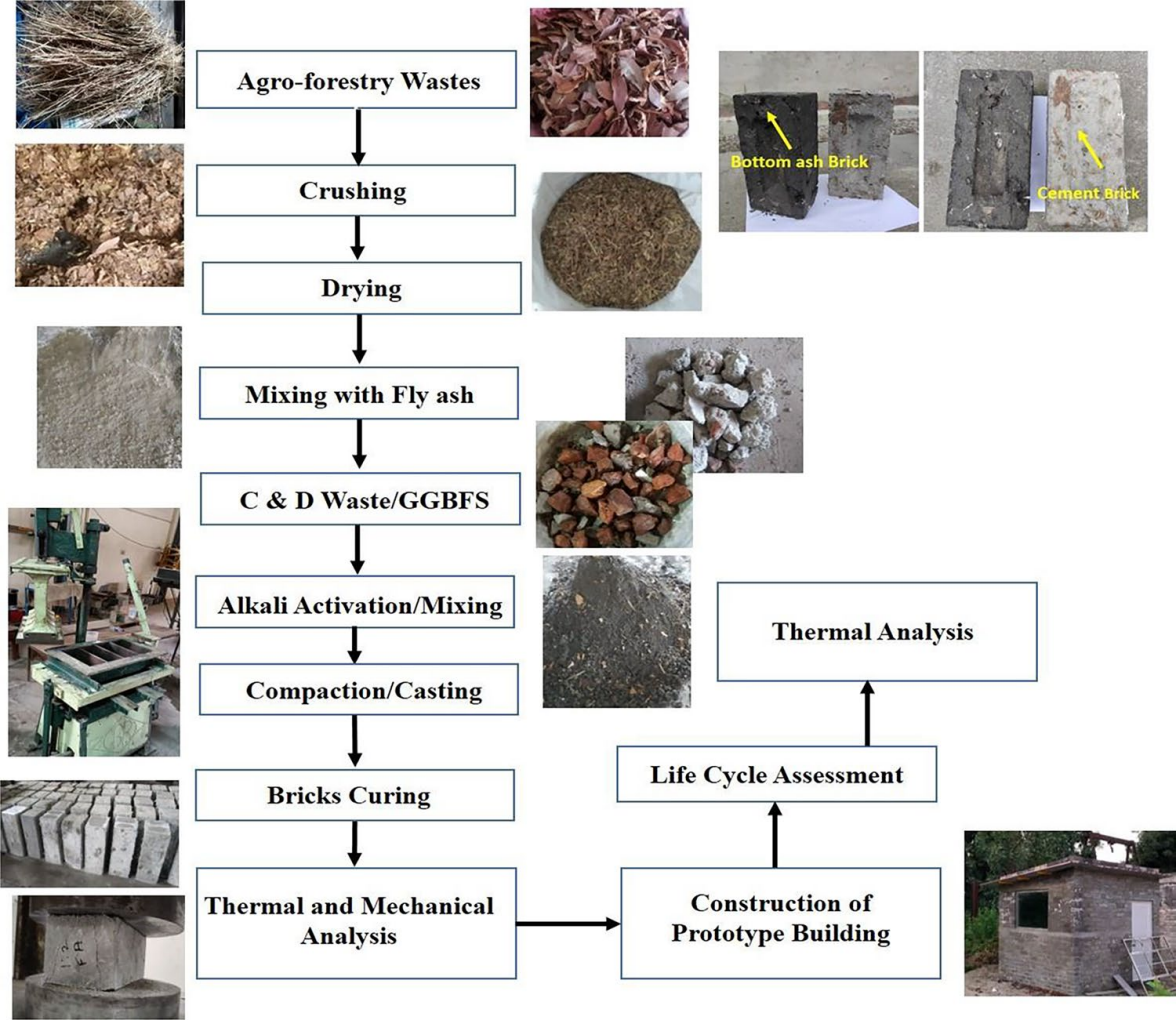
Process methodology for agro-forestry waste based fly ash, bottom ash and cement based bricks using vibro-compaction technique.

Bricks were produced by a indigenously developed brick-making machine based on vibro-compaction technique employing a four-bar slider-crank linkage configuration based ejection mechanism for demolding, and a plunger weight of 15 kg for compaction with the options of 4–8 bricks per batch^33^. The dimension of the casted bricks was 230 mm × 110 mm × 70 mm. The raw materials utilised in brick casting are agro-forestry waste 2 wt% (of fly ash), GGBFS: 10 wt%, C&D waste: 15–20 wt% with approximate size of 50–80 mm × 40 mm × 40 mm with 3 M NaOH as activator solution. The ratio of fly ash to fine aggregate (sand) was in the ratio of 1:2. For comparison purpose, cement based (OPC-43 grade) and bottom ash bricks were also casted and tested for mechanical properties. The casted bricks were cured at ambient temperatures of around 30–32 °C for 28 days. The various steps involved for brick casting process are shown in Fig. 1. The nomenclatures of the investigated bricks are: *A- cement based bricks, B- fly ash based bricks, C- bottom ash based bricks*. The bottom ash collected from the same source has similar chemical constituents with a majority part of unburnt carbon residue/soot (black colour), which is a distinguished feature of bottom ash in physical appearance which was also visible in casted brick samples. The mix design parameters of the casted fly ash, cement and bottom ash bricks are shown in Table 2.

**Table 2:** Mix design parameters of casted bricks.

Mix ID	Binder	Agro-forestry waste (wt%)	C&D waste (wt%)	Slag (wt%)	Binder**:**Sand	Water**:**Binder
Fly ash Bricks	Fly ash	2	15–20	10	1:2	0.4–0.5
Cement Bricks	Cement	2	15–20	10	1:2	0.45
Bottom ash Bricks	Bottom ash	2	15–20	10	1:2	0.6–0.8

### Characterization

The elemental, compositional and quantitative analysis of fly ash, GGBFS, rice straw and forestry leaves have been mentioned in Table 1 and detailed study in the authors’ previous publications^26,32^ . The mechanical performance of the alkali activated fly ash (AA-FA) bricks was analysed by compressive strength test in accordance with relevant Indian standard codes^34–36^. The brick specimens have been tested on a universal testing machine (UTM) of 100-ton capacity (Shimadzu) with a loading rate of 140 kg/cm^2^/min as specified in IS code^36^.

### Construction of prototype building

**Figure 2:**
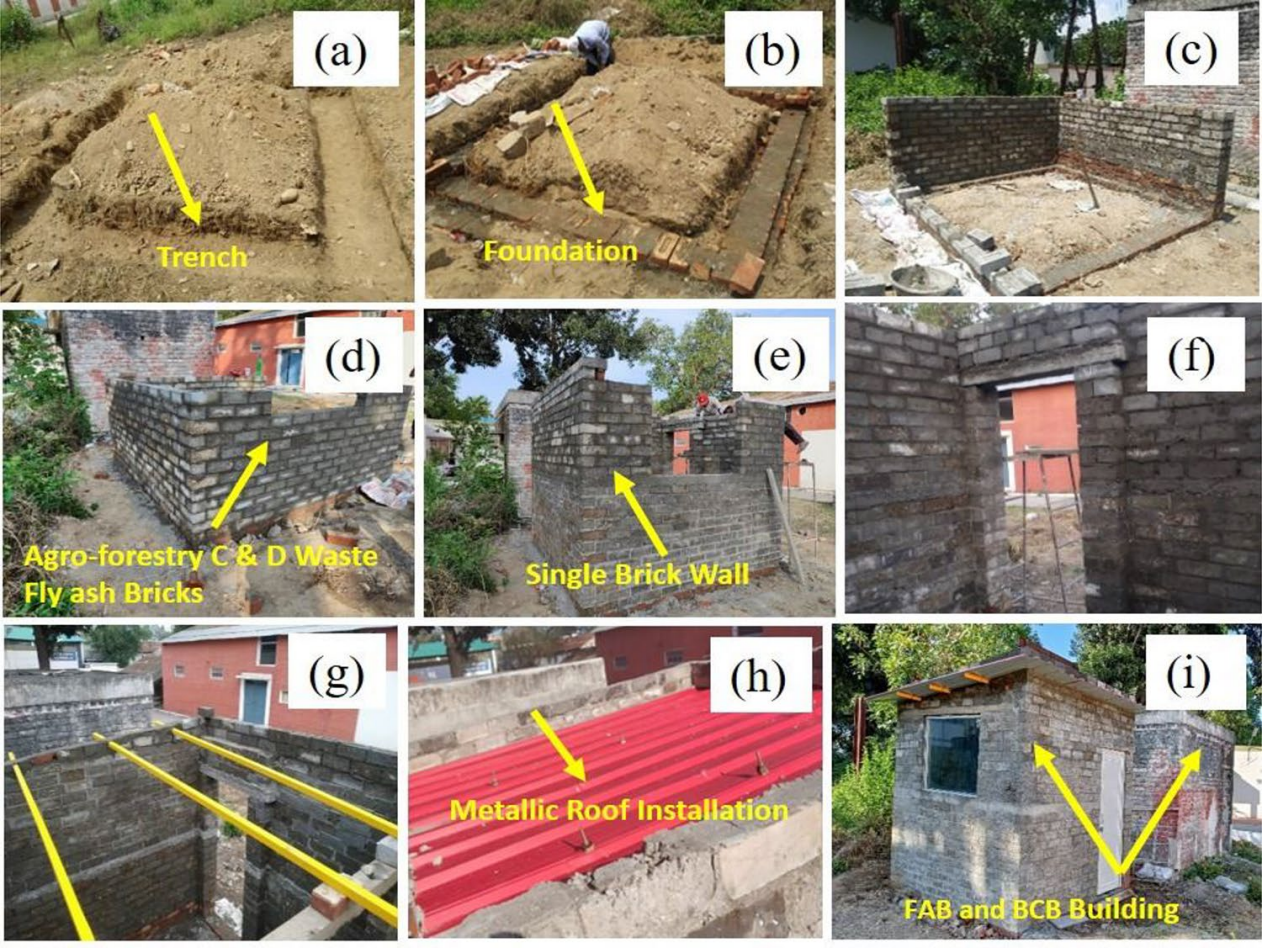
Foundation laying and construction of fly ash based bricks building.

A prototype building comprising of single room was constructed from agro-forestry waste, C&D waste based fly ash based bricks with dimensions 8 ft. × 9 ft. × 8 ft. to investigate the feasibility of bricks in non-load bearing application and evaluation of thermal and carbon emission parameters. The foundation of the building was 1 ft. × 1 ft. in width and depth laid on burnt clay bricks to provide additional strength to the foundation. The prototype building was fitted with a 3 ft. × 4 ft. double-glazed glass window and 6 ft. × 2.5 ft. door with a metallic sheet roof. The whole construction process starting from foundation to roof installation is shown in Fig. 2. A total of 1000 bricks comprised of 1000 kg of fly ash, 2000 kg of fine aggregate (sand), 150–200 kg of C&D waste, 20–25 kg of agro-forestry waste, and 100 kg of GGBFS as waste material was utilised in construction of prototype building.

### CO_2_ emission analysis by LCA approach and embodied energy estimation

The environmental concern of developed fly ash bricks in terms of CO_2_ emission and its comparison with burnt clay bricks has been done in the present study by LCA approach analytically for cradle to gate cycle assessment. The calculations and analysis have included the resources and processes involved in manufacturing of the bricks. The various stages starting from extraction, transportation of raw materials to factory site, firing/casting, storage and distribution causing CO_2_ emission has been taken into consideration in the present study. Basic CO_2_ emission calculations for manufacturing burnt clay and fly ash bricks at various stages have also been discussed in subsequent sections.

#### CO_2_ emission analysis of burnt clay bricks

The various stages in LCA analysis of burnt clay bricks include extraction of raw materials, manufacturing/firing, and storage/distribution.

#### Emission due to raw material extraction

In Indian scenario, the excavation and extraction of soil for brick manufacturing is labour intensive. According to the specification laid for the assessment of the greenhouse gases emission of goods and services of human energy involvement is excluded. Therefore, ‘soil excavation’ process is not included in CO_2_ emission and hence no estimation is required^37^.

#### Emission due to raw material transportation

The calculation of carbon emission involved in material transportation, is done by the “ton-kilometer” (measured in t-km) estimation. The ton-kilometer is the measurement for the transportation of 1-ton of a material by a given transport mode over a distance of 1 km. Subsequently, the CO_2_ emission due to transportation is then calculated by multiplying the value of t-km by the emission factor (in kgCO_2_/t-km) and the total mass of material transported^38,39^.

In Indian scenario, usually the clay field and the brick kiln is situated within the distance of 1–2 km. For 1-kg of brick manufacturing, ~ 1.11 kg of clay is required. Therefore, the t-km estimation for 2-km distance is

1 $$2 \times \frac{1.11}{1000}=0.0022\, {\text{t}}-{\text{km}}$$

The average gCO_2_ emission of 40-ton truck/tractor is 62 g CO_2_/t-km^38^. By multiplying the above value with the gCO_2_ emission factor of 40-ton truck/tractor, the total CO_2_ emission per kg of brick is obtained as:

2 $$0.0022 \times 62=0.136\,\mathrm{g}{{\text{CO}}}_{2}$$

#### Water requirement scenario

The transported clay which has been brought to manufacturing site situated near brick kiln location is kept and stored in pile form. The stored clay does not require any further additives prior to casting. The water requirement for clay brick casting is around 20–30%, usually taken from rivers, ponds or bore well pumped by 2–3 hp motor. On an average, the electrical power and CO_2_ emission factor associated with the electricity generation and transmission for desired amount of water for 100,000 bricks casting is around 18.77 kWh, 1.36 kgCO_2_/kWh and 0.47 kg CO_2_/kWh respectively^40^.

For 1-brick, the CO_2_ emission due to water requirement is given as

3 $$0.0136 +0.0047)/2.8 =0.006\,\mathrm{g}{{\text{CO}}}_{2}$$

Post green brick casting, the bricks are sundried for about 10–15 days prior to firing in a kiln. Majority of the brick kilns are coal and bagasse fired. The firing process is a major cause of CO_2_ emission in clay bricks production.

#### Fuel transportation

The transportation of coal as fuel in brick kilns is done via. Rail routes in India from coal mines/fields. Then it is locally distributed to the nearby regions of brick kilns via road transports.

According to the literature^38,39^, the average CO_2_ emission per ton-km for coal transportation from an electric locomotive goods trains is 22 gCO_2_ per t-km.

Average ton-km calculation for 70 gm coal transported to a distance of 1000 km is:

4 $$1000 \times \frac{0.07}{1000}=0.07\, {\text{t}}-{\text{km}}$$

Therefore, the average CO_2_ emission for fuel transport via rail route is:

5 $$0.07\times 22=1.54\,\mathrm{g}{{\text{CO}}}_{2}$$

Ton-km estimation for coal as fuel transport from railway wagons to local kilns situated at a distance of 50 km is:

6 $$50 \times \frac{0.07}{1000}=0.0035\, {\text{t}}-{\text{km}}$$

Average gCO_2_ emission of 40-ton truck/tractor for coal transportation is given as:

7 $$0.0035 \times 62=0.217\,\mathrm{g}{{\text{CO}}}_{2}$$

#### Brick firing and CaCO_3_ decomposition from soil

The CO_2_ emission from coal firing for 1-kg of brick is ~ 64.26 gCO_2_. During the firing of bricks, the calcium carbonate (CaCO_3_) present in the clay decomposes into calcium oxide (CaO) and CO_2_ resulting in emission of 25.9 gCO_2_/kg of fired brick^40,41^.

#### Storage and distribution

After firing process, the bricks are stored in the vicinity areas for storage and distribution. For a particular kiln site, the distribution area is situated within the range of 25–30 km.

The ton-km estimation of transporting 1 kg of brick to a distance of 30 km can be calculated as:

8 $$30 \times \frac{1}{1000}=0.03\, {\text{t}}-{\text{km}}$$

The average gCO_2_ emission of 40-ton truck/tractor is 62 g CO_2_/ton-km. the total CO_2_ emission per kg of brick distribution to a distance of 30 km is obtained as:

9 $$0.03 \times 62=1.86\, {{\text{gCO}}}_{2}$$

#### Total estimation of carbon emission for burnt clay bricks

For total CO_2_ emission estimation, overall CO_2_ emissions due to all the operations involved in the burnt clay brick manufacturing can be summarized as:

10 $$\sum_{i=1 }^{n}{Activity}_{{\text{t}}-{\text{km}}} \times {Emission\, factor}_{{{\text{gCO}}}_{2}per {\text{t}}-{\text{km}}}$$

The CO_2_ emission norms from mini diesel mini in India, according to international council on clean transportation is 143.1 gCO_2_/km, with load carrying capacity of 1 ton and 0.011 gCO_2_/t-km for electric locomotive (freight trains) in India^42,43^.

### CO_2_ emission analysis of agro-forestry waste fly ash bricks

#### Emission due to raw material transportation and NaOH

In the present study, fly ash as raw material is transported from Dadri power plant (Uttar Pradesh, India). The ton-km estimation for 180 km distance and 1 kg of fly ash is given as

11 $$180 \times \frac{1}{1000}=0.18\, {\text{t}}-{\text{km}}$$

By multiplying the above value with the emission of 40-ton truck, the total CO_2_ emission per kg of fly ash is obtained as:

12 $$0.18 \times 62=11.16\,\mathrm{g}{{\text{CO}}}_{2}$$

The CO_2_ emission due NaOH production results in 1.915 ton-CO_2_ emission per ton (of NaOH produced). For 3 M solution used in brick casting with water: binder ratio of 0.3, the emission due to NaOH utilisation is around 20.56 gCO_2_. The emission due to GGBFS utilisation may be considered to 0.52 gCO_2_.

#### Water requirement scenario

The water requirement for agro-forestry and C&D waste fly ash bricks in the present study is in the range of 0.4–0.5. The CO_2_ emission due to water pumping (required for 1-kg of brick) is taken similar to the clay brick which is given as:

13 $$(0.0136 +0.0047)/3 =0.006\, {{\text{gCO}}}_{2}$$

#### Emission due to C&D waste generation and transportation

The C&D waste generated from demolition of buildings, structures etc., requires a diesel powered equipment. The approximate value of energy consumption is 0.0359 MJ of diesel per kg of brick demolished. The energy content of diesel is 38.29 kJ/litre^44^. The energy required for 1-kg of brick demolition is 35.9 kJ which consumes 0.93 L of diesel. The C&D waste generated from the site is transferred to the brick casting site within 2 km range and crushed manually to the desired size in the present study.

The ton-km estimation for transporting 1-kg of C&D waste to a distance of 2 km is given as

14 $$2 \times \frac{1}{1000}=0.002\, {\text{t}}-{\text{km}}$$

The average gCO_2_ emission of 40-ton truck to carry 1 kg of C&D waste as described by:

15 $$0.002 \times 62=0.124\,\mathrm{g}{{\text{CO}}}_{2}$$

1-brick utilises approximately, 150–200 gm of coarse C&D waste, therefore net CO_2_ emission will be 0.024 ${{\text{gCO}}}_{2}$.

#### Emission due to agro-forestry waste transportation

The transportation of agro-forestry wastes is usually done within 10 km range. The ton-km estimation is given as

16 $$10 \times \frac{1}{1000}=0.01\, {\text{t}}-{\text{km}}$$

The average gCO_2_ emission of 40-ton truck to carry 1 kg of agro-forestry waste is:

17 $$0.01 \times 62=0.62\,\mathrm{g}{{\text{CO}}}_{2}$$

1-brick utilises approximately, 10 gm of agro-forestry waste, therefore net CO_2_ emission will be 0.6E-6 ${{\text{gCO}}}_{2}$.

#### Emission due to fine aggregate (sand) transportation

The fine aggregate (sand) is transported from nearby river bank situated around 80 km from the casting site. The ton-km estimation is given by:

18 $$80 \times \frac{1}{1000}=0.08\, {\text{t}}-{\text{km}}$$

The average CO_2_ emission per kg of material by 40-ton truck is given as:

19 $$0.08 \times 62=4.96\,\mathrm{g}{{\text{CO}}}_{2}$$

1-brick utilises 2 kg of fine aggregate, therefore net CO_2_ emission will be 9.92 ${{\text{gCO}}}_{2}$.

#### Electricity requirement scenario

The electricity requirement for brick manufacturing by the indigenous brick making machine utilizing 3 hp motor is ~ 458 kWh (for 100,000 bricks, vibration and loading for 30 s) resulting in CO_2_ emission 1.36 kg CO_2_/kWh and 0.47 kg CO_2_/kWh for generation and transmission, respectively.

The CO_2_ emission for electricity generation and transmission per kWh is 1.83 gCO_2_.

Therefore, the CO_2_ emission for 100,000 brick casting using electric brick making machine is given by:

20 $$1.83{{{\text{gCO}}}_{2}}_{2}/{\text{kWh}} \times 2.2 {\text{kW}} \times 208 {\text{h}} =838.74\, {{\text{gCO}}}_{2}$$

Therefore, 01 brick casting would result in 0.008 gCO_2_ emission.

#### Curing of bricks

The casted bricks are dried/cured at ambient temperature for 28 days for optimum strength gain and does not require any firing process.

#### Storage and distribution

The ton-kilometer estimation of transporting 1 kg of brick to a distance of 30 km, can be calculated as:

21 $$30 \times \frac{1}{1000}=0.03\, {\text{t}}-{\text{km}}$$

The total CO_2_ emission per kg of brick distribution to a distance of 30 km is obtained as:

22 $$0.03 \times 62=1.86\,\mathrm{g}{{\text{CO}}}_{2}$$

1-brick weighs approximately, 3–3.2 kg, therefore net CO_2_ emission will be 5.95 ${{\text{gCO}}}_{2}$.

#### Total CO_2_ emission estimation of fly ash bricks

For total estimation of carbon emission by fly ash bricks, total CO_2_ emissions due to all the operations involved in the fly ash bricks production can be summarised as:

23 $$\sum_{i=1 }^{n}{Activity}_{{\text{t}}-{\text{km}}} \times {Emission\, factor}_{{{\text{gCO}}}_{2}per {\text{t}}-{\text{km}}}$$

Embodied energy calculation in the present study have been done using LCA approach along with inventory for carbon and energy database. The embodied energy may be defined as total primary energy (e.g., coal; energy extracted form nature) utilised from direct and indirect processes associated with a product or services within the boundaries of cradle-to-gate. This boundary approach includes the manufacturing process starting from raw material extraction, transportation and manufacturing until the end product is ready to leave the factory gate^45^. Embodied energy has a great importance in the context of buildings and construction materials because a high energy intensive material is more likely to cause larger carbon emission.

### Thermal analysis

#### Steady State thermal properties

The thermo-physical properties viz., thermal conductivity (*k*), specific heat capacity (*C*_*p*_) and density (*ρ*) are basic thermal properties of any building materials. The thermal properties derived from the basic properties are thermal transmittance (U), thermal diffusivity (α), thermal effusivity (τ), and thermal mass (m) are equally important for thermal properties evaluation. Steady-state thermal properties of building envelope were calculated according to Indian standard IS 3792–1978^46^.

The constructed agro-forestry waste based fly ash bricks and burnt clay brick walls of the respective buildings were tested for temperature variations on inner and outer wall surfaces, ambient and inside room temperature for a period of 24 h and 07 days for June month. The instrument used for wall temperature measurement was an infrared thermometer Fluke 64 MAX having a temperature range of − 30 °C to 600 °C with an accuracy of ± 1.5 °C. According to guide for heat insulation of non-industrial buildings IS 3792–1978, no heating or cooling aids were used during the measurement. The thermal transmittance (U-value) of fly ash brick wall and burnt clay brick wall envelope was measured by Testo 635 U-value probe meter based on the formula:

24 $$U= \alpha \frac{{T}_{in}-{T}_{surface, in}}{{T}_{in}-{T}_{out}}$$

where, *α* is 7.69 W/m^2^K, *T*_*in*_ inside room temperature, *T*_*surface, in*_ is inside room surface temperature, *T*_*out*_ is outside ambient temperature.

#### Dynamic thermal properties

Non-steady state thermal properties viz., time lag, decrement factor, thermal damping and thermal admittance are dynamic thermal properties which provide real time assessment of building envelopes. In this study, the thermal performance of building walls has been investigated by admittance method.

## Results and discussion

### Mechanical properties of fly ash based bricks

**Figure 3:**
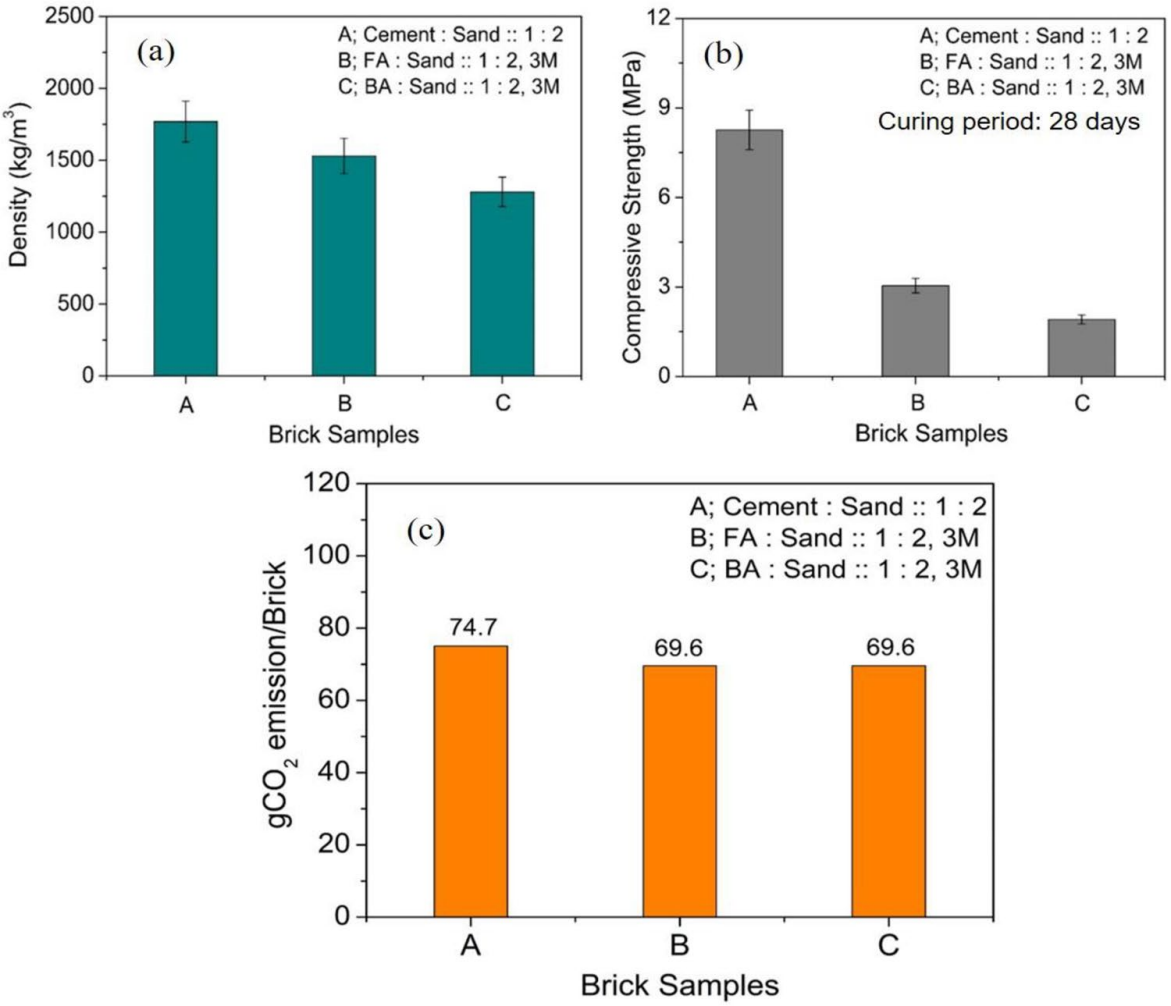
Mechanical aspects of agro-forestry waste based cement, fly ash and bottom ash bricks (**a**) Density and (**b**) Compressive strength variation of bricks for curing of 28 days, (**c**) CO_2_ emissions analysis.

Authors’ previous study has explored the utilisation of alkali-enzyme treated agro-forestry residues along with C&D waste in fly ash bricks^26^. In the present study, a simple approach has been adopted for agro-forestry waste utilisation. The mixture of untreated rice straw and dried forestry leaves (2 wt% of fly ash) crushed and chopped to desired size mixed in fly ash and sand along with C&D waste. Figure 3a,b show the density and compressive strength values of 2-wt% added agro-forestry waste and C&D waste fly ash bricks along with bottom ash and cement based bricks. The cement and bottom ash based bricks investigated in the present study are only for comparison purpose. The density of these bricks shows a reducing trend with values ranging from 1400 to 1600 kg/m^3^ as shown in Fig. 3a. The compressive strength of the bricks lies in the range of 1.5–8 MPa with lowest value corresponding to bottom ash bricks and 3–4 MPa for fly ash based bricks and maximum for cement based bricks shown in Fig. 3b. The cement based bricks have highest density and compressive strength due to better formation of hydration products viz., calcium silicate hydrate (C–S–H).

For fly ash based bricks, the reduction in compressive strength as compared to authors’ previous study may be attributed to low degree of formation of hydration products viz., sodium aluminate silicate hydrate (N-A-S–H) or calcium/sodium aluminate silicate hydrate (Ca–Na)–A–S–H gel. The reduced formation of the hydration products may be attributed to low molarity of NaOH solution (3 M) which results in lesser dissolution of aluminosilicates to release SiO_4_ and AlO_4_ tetrahedral units responsible for polycondensation process. The addition of GGBFS in the mix design partially substitutes Na ions with Ca ions resulting in formation of (Ca–Na)–A–S–H gel with quick hardening of the mix at ambient temperatures^47,48^. The authors’ detailed previous study has shown the effect of slag addition, NaOH molar concentration, average particle size on physical properties viz., compressive strength, density, porosity with varying slag content in fly ash bricks^26^. The bottom ash bricks show poor compressive strength due to the presence unburnt carbon as agglomerates resulting in coarser particles (~ 150 µm) which limits the reactivity of alkaline solution and dissolution of aluminosilicates in the mix. The bottom ash absorbs most of the alkali solution, which increases the liquid to solid ratio with reduced reactivity. The quality of the bottom ash can be further improved by mechanical activation process, through combustion to reduce the LoI/unburnt carbon content which will result in enhanced reactivity. But the process of making bottom ash reactive is energy intensive and has environmental issues related to carbon emission^49^.

The particle size and shape of slag and fly ash play an important role as the two parameters are responsible in determining the chemical reactivity leading to enhanced physical parameters. The fly ash utilised from Dadri power plant and GGBFS have an average particle size of 25.6 µm and 25.1 µm respectively. The smaller particle size results in higher surface area which increases the overall reactivity at solid–liquid interface. According to the literature, the smaller size particles have greater amorphous content as they quench more quickly than larger particles^50^. The x-ray photoelectron spectroscopy (XPS) analysis of the various fly ash done in the authors’ previous study has able to identify the major constituents and their amorphous phases present in the fly ash samples. The amorphous (reactive) content of silica present in fly ash responsible for reactivity with alkali solution resulting in polycondensation process has been studied in detail^32^. Furthermore, the spherical shape of particles improves the workability of the mix at lower liquid to binder ratio resulting in less water requirement. The thermal conductivity data for 25 mm thick agro-forestry based fly ash sample shows the value over the range of 0.4–0.45 W/m K^26^. The burnt clay brick sample has the thermal conductivity value of 0.8 W/m K, which is almost double the value of fly ash brick samples. The enhanced value of thermal insulation of fly ash brick samples will provide better thermal insulation and comfort to the building occupants. A brief carbon emission analysis of cement, fly ash and bottom ash bricks is shown in Fig. 3c. From the graph 3(c), it is evident that even fly ash bricks with alkali activation shows moderate CO_2_ emission as compared to cement based bricks. This is attributed to high CO_2_ emission associated with NaOH production which is nearly 1.915 ton-CO_2_/ton NaOH produced. The overall emission factors have been taken from Table 3 regarding brick manufacturing.

**Table 3:** CO_2_ emission for burnt clay and fly ash bricks at various production stages.

CO_2_ emission stages	gCO_2_ emission burnt clay brick (with European emission values)	gCO_2_ emission fly ash brick (with European emission values)	gCO_2_ emission burnt clay brick (with Indian emission values)	gCO_2_ emission fly ash brick (with Indian emission values)
Extraction	Nil	Nil	Nil	Nil
Material Transportation	0.136	16.77	0.3146	48.61
Water, NaOH & GGBFS	0.006	20.56 & 0.52	0.006	20.56 & 0.52
Fuel Transport	1.757	Nil	0.50	Nil
Firing/Electricity	90.16	0.008	90.16	0.008
Storage and Distribution	5.02	5.95	11.59	13.73
Total CO_2_ @1 kg of brick	97.08	14.6	102.07	27.63
Total CO_2_ @ 1 brick	290	43.80	285.6	82.90

### Carbon emission estimation by LCA approach

#### Total CO_2_ emission analysis of burnt clay bricks

**Figure 4:**
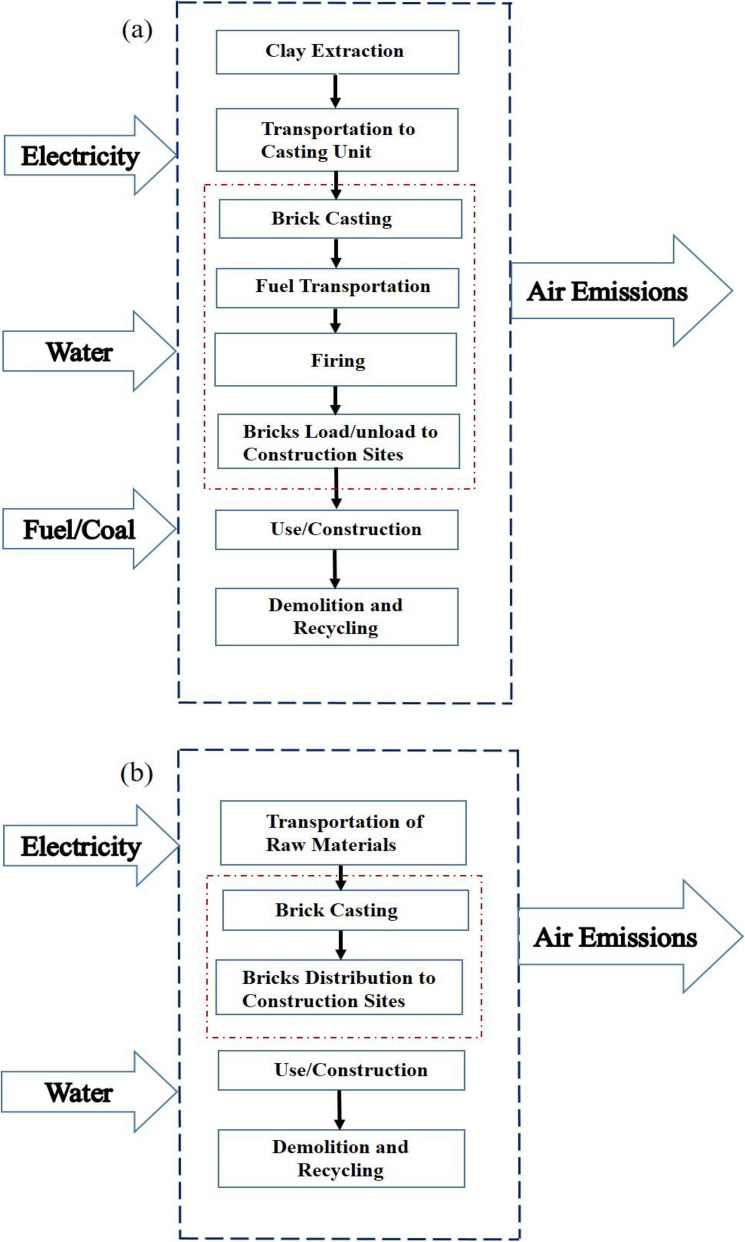
System boundary conditions for (**a**) Burnt clay bricks and (**b**) Fly ash bricks. Outer enclosed structure shows system boundary (blue colour) and inner enclosed structure shows production stage (red colour).

Post mechanical analysis of fly ash bricks, the environmental impact analysis of fly ash bricks by LCA approach and its comparison with burnt clay bricks has been investigated in this section. The various stages in LCA analysis of both the type of bricks include extraction of raw materials, manufacturing/firing, distribution, demolition, and end of life cycle. A diagram showing the system boundary for process illustration for burnt clay and fly ash bricks production is shown in Fig. 4. The diagram shows outer enclosed structure as system boundary and red line in interior part as production stage of the bricks.

For total estimation of CO_2_ emission for burnt clay bricks, overall CO_2_ emissions due to all the operations involved in the burnt clay brick manufacturing as mentioned in detail in Section "CO2 emission analysis by LCA approach and embodied energy estimation" is considered. According to Eq. (10), the overall carbon emission values for 1 kg of brick production is 97.08 gCO_2_ and 270 gCO_2_ per brick according to European emission values^38,39^.

The CO_2_ emission from diesel mini trucks in India, according to international council on clean transportation is 143.1 gCO_2_/km, with load carrying capacity of 1 ton and 0.011 gCO_2_/t-km for electric locomotive (freight trains) in India^42,43^.

By substituting the above emission values, the overall CO_2_ emission of burnt clay bricks manufactured according to Indian emission norms is approximately 102 gCO_2_ per kg of clay brick.

Average weight of 1 burnt clay brick is ~ 2.8 kg. Therefore, total ${{\text{CO}}}_{2}$ emission for 1 brick ~ 285.6 gCO_2_ according to Indian transport emissions of vehicles and freight trains. The complete CO_2_ emission in burnt clay bricks production is summarised in Table 3.

#### Total CO_2_ emission analysis of agro-forestry waste fly ash bricks

For total estimation of carbon emission by fly ash bricks, total CO_2_ emissions due to all the stages considered in fly ash bricks production can be calculated according to Eq. (23) as 43.28 gCO_2_ per brick. The total CO_2_ emission for 1000 fly ash bricks is 38.26 kgCO_2_ in construction of prototype single room masonry building in the present study.

The CO_2_ emission from diesel mini trucks per km in India, according to ICCT is 143.1 g CO_2_/km, considering load carrying capacity of 1-ton^42^. The values of carbon emission either by passenger or goods vehicles vary because there is no official breakdown of road fuel use data by various vehicle types in India. For majority of Asian countries, there are also no published data on vehicle-km or ton-km for goods vehicle by the main modes of transportation. Therefore, it is not possible to estimate CO_2_ emissions for each major activity within the transport sector^51^. By putting the above emission values, the overall CO_2_ emission of agro-forestry waste based fly ash bricks manufactured according to Indian emission norms is approximately 27.63 gCO_2_ per kg of brick. Weight of 1 fly ash brick is ~ 3.0 kg. Therefore, total emission for 1 brick as per Indian emission values ~ 82.89 gCO_2_.

**Figure 5:**
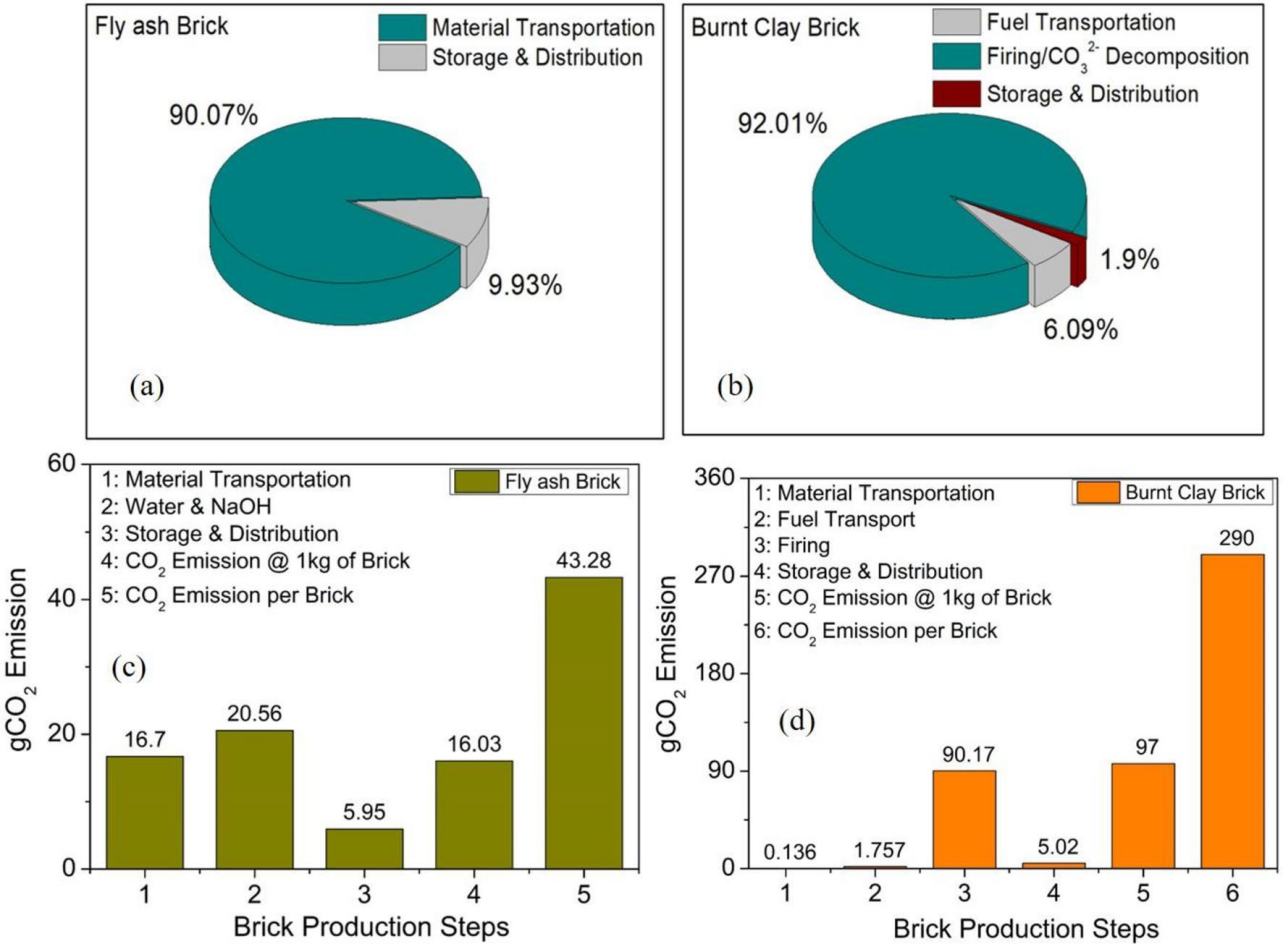
Carbon emission at various stages of bricks production. Percentage wise CO_2_ emission for (**a**) Fly ash and (**b**) Burnt clay bricks, (**c**) and (**d**) CO_2_ emission from at various production stages of fly ash and burnt clay bricks.

The CO_2_ emission values at various stages for burnt clay and fly ash bricks with both (European and Indian) emission values is summarised in Table 3. The CO_2_ emission from major steps and their percentage distribution of fly ash and burnt clay bricks is shown in Fig. 5c,d. Approximately, 90% of carbon emission in fly ash brick production arises from material transportation and demolition activities (Fig. 5a) and 10 percent from distribution activity. Similarly, for burnt clay bricks production, the major portion (around 92%) of carbon emission is due to firing process and carbonates decomposition of carbonates present in the clay/soil (Fig. 5b).

Net CO_2_ reduction of utilizing agro-forestry waste and C&D waste based fly ash bricks when compared with burnt clay bricks is ~ 246 gCO_2_ per brick as per European emission norms. According to a literature study, the overall CO_2_ emissions in manufacturing of burnt clay bricks in clamps is around 195 gCO_2_/kg of fired brick^40^. In the present study, the overall CO_2_ emission for burnt clay bricks is around 102 gCO_2_/kg of brick calculated with Indian emission values. From Table 3, it is evident that carbon emission values of burnt clay bricks are almost 4–8 times higher than carbon emission of fly ash bricks with a similar trend in embodied energy values described in next section. A similar study on utilisation of boiler ash collected from pulp and paper mills with alkali activation in masonry construction materials has been reported. The obtained ash mixed with clay and lime activated with 2 M NaOH solution exhibits compressive strength in the range of 11–16 MPa cured at 30 °C for 28 days. The compressive strength of fly ash bricks with 3 M NaOH activation in the present study lies in range of 3–6 MPa. The environmental impact assessment of boiler ash shows Ca(OH)_2_ and NaOH as biggest CO_2_ emitters (30–65% of total emission) as their production is energy intensive and 90% of CO_2_ emission for burnt clay bricks during firing process. The values reported in the literature is in agreement with the carbon emission values obtained in the present study. Another similar study regarding environmental assessment on rice husk ash incorporation in fly ash bricks with alkali activation shows almost 60–90% of environmental impact is due to manufacturing of raw materials^49,52^.

#### Embodied energy estimation of burnt clay and fly ash bricks

In the present study, the embodied energy of a typical building/construction materials may be defined as overall primary energy consumed starting from raw material extraction, manufacturing, storage and transportation till the material is ready for distribution at the factory gate. This type of approach is usually termed as cradle to gate approach which is employed in the embodied energy estimation. Table 4 shows the embodied energy of both burnt clay bricks and fly ash bricks constituents and total energy consumed^45^. From the estimation it is clear that burnt clay bricks are 10–15 times more energy intensive compared to fly ash bricks which consumes more energy during the firing process resulting in CO_2_ emission as evident from LCA analysis done in previous section.

**Table 4:** Embodied energy estimation of fly ash and burnt clay bricks^45^.

Materials	Embodied energy (EE) (MJ/kg)		Required EE (MJ/kg)
	Burnt clay brick	Fly ash brick	Fly ash brick
Clay	3.0	–	–
Fly ash	–	0.1	0.1
Fine aggregate	–	0.08	0.16
Coarse aggregate	–	0.083	0.03
GGBFS (Slag)	–	1.6	0.16
Water	0.01	0.01	0.01
Total	6.9 (2.3 kg per brick)		0.46 (3.0–3.2 kg per brick)

Considering the current requirement of clay bricks in India which is around 250 billion bricks per year, the overall carbon emission values would be 72.5 billion tons CO_2_. According to a report published by CEA (2022), the total utilisation of fly ash in manufacturing of bricks and tiles is 31.62 million tons which is 11.68% of total fly ash utilisation and 25.41% is utilised by various cement industries. Out of total fly ash generation of 270.82 million tons from thermal power plants, still approximately 11 million tons of fly ash is non-utilised^9^. The extraction of top fertile soil for burnt clay brick production causes land degradation resulting in reduced soil carbon content, microbial activity and nitrous oxide concentrations. The consequence of this activity may alter the CO_2_/GHGs concentration in the atmosphere to a great extent with increase in global temperature and change in weather patterns. Whereas, the fly ash with properties viz., odorless, non-toxicity, non-flammability with minimum threat to human health may serve as potential substitute for burnt clay bricks. Fly ash based bricks have low water permeability, shrinkage, enhanced workability, high thermal insulation and chemical resistance towards sulphates and chlorides and excellent finishing. All these features of fly ash based bricks makes it economical and a potential green building material along with other incorporated wastes. The cost of the fly ash bricks varies with many parameters viz., material cost, quality, transportation charges and manpower charges. In the present case, the cost of one fly ash brick is approximately ₹3–6 considering all the costs including material transportation and electricity consumption. The price of one burnt clay brick also varies from region to region with similar factors as mentioned for fly ash bricks.

**Figure 6:**
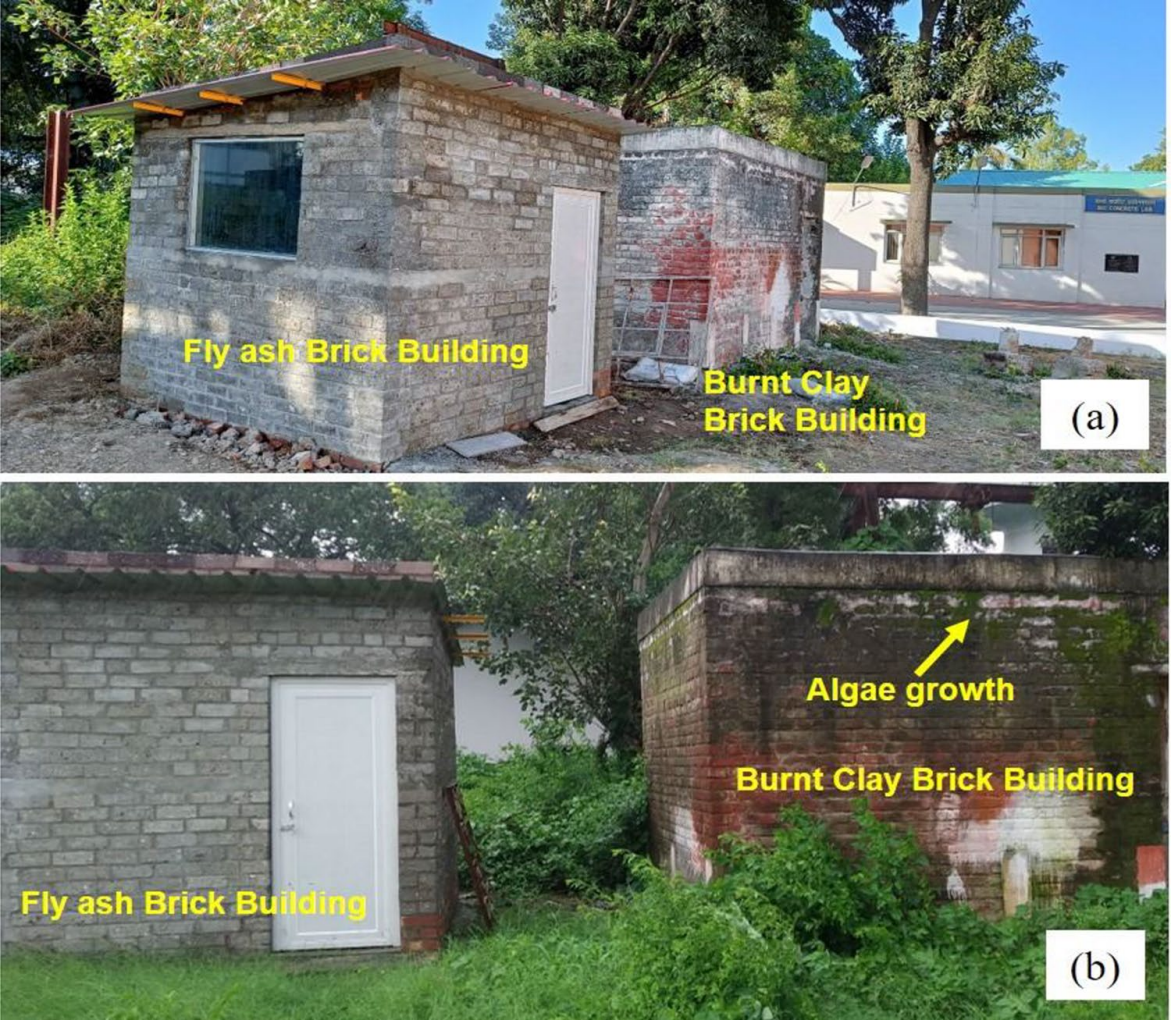
Physical appearance of fly ash and burnt clay bricks building during (**a**) Summer season, (**b**) Monsoon (rainy) season.

After mechanical, thermal and LCA analysis, a brief investigation of moisture and algae growth on fly ash brick and burnt clay brick building has been done during monsoon to assess the suitability of fly ash bricks for outdoor application. Figure 6 shows the exterior walls of both the buildings exposed to seasonal rainfall (monsoon period) for the period of June to July. The average moisture content of agro-forestry waste based fly ash brick building is 25–40%. Whereas, the moisture content for burnt clay brick building lies in the range of 40–50% which shows higher water absorption of burnt clay bricks. The exterior walls of both the buildings for the summer season is also shown in Fig. 6a, where no algae or fungal growth are observed. From Fig. 6b, algae and fungal growth during monsoon season on external brick wall of burnt clay brick building can be clearly seen which may lead to deterioration of wall surfaces. Whereas, no degradation or visual damages are observed for fly ash brick building due to their low water absorption and chemically (alkali) treated bricks.

### Thermal analysis of fly ash and burnt clay brick buildings

#### Steady state thermal analysis

**Figure 7:**
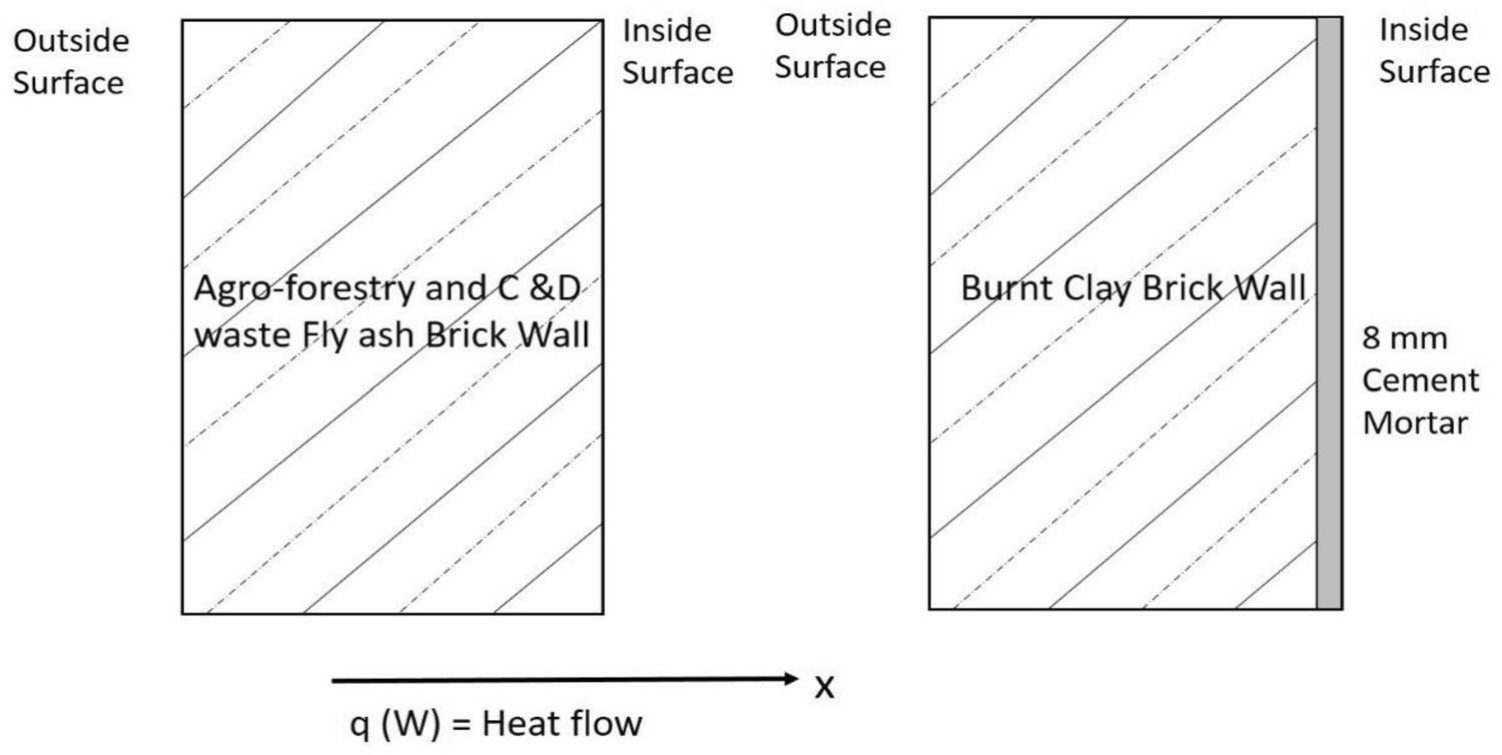
Schematic wall configurations of fly ash and burnt clay brick walls.

The aim of studying thermal properties of building materials is to investigate the indoor performance and thermal comfort of the buildings. Steady state parameters viz., thermal conductivity, thermal transmittance (U-value), thermal diffusivity, specific heat and thermal effusivity are determined and compared for fly ash and burnt clay bricks. The investigated building walls are shown schematically in Fig. 7 and wall dimensions, properties are mentioned in Table 5.

**Table 5:** Features of fly ash and burnt clay brick buildings.

Features	Building dimensions (in feet)	Wall thickness (mm)	Sp. heat capacity	Ceiling/roof	Window
Fly ash bricks	8 ft. × 9 ft. × 8 ft	70	930	Metallic	Double glazed
Burnt clay bricks	8 ft. × 10 ft. × 10 ft	230	880	Concrete	–

The one dimensional (1-D) heat transfer process may be written as:

25 $$\frac{\partial T}{\partial t}=\frac{\lambda }{{\rho c}_{p}} \frac{{\partial }^{2}T}{{\partial x}^{2}}$$

where, $\lambda$ is the thermal conductivity, *ρ* is the density, and *c*_*p*_ is the specific heat capacity of the building wall materials.

The Eq. 25 can be rewritten as:

26 $$\frac{\partial T}{\partial t}=\alpha \frac{{\partial }^{2}T}{{\partial x}^{2}}$$

where, $\alpha$ is the thermal diffusivity defined as ($\lambda$/*ρ. C*_*p*_) and expressed in m^2^/s. The thermal diffusivity may be defined as rate at which thermal disturbance propagates and is a thermo-physical property of the material.

The thermal effusivity which is ability of material to exchange heat with the surroundings can be written as,

27 $$\xi = \sqrt{\lambda .\rho . c}$$

where as, $\xi$ is thermal effusivity in J m^−2^ K^−1^ s^0.5^, *ρ* is the density of the material, *C*_*p*_ is specific heat capacity in J Kg^−1^ K^−1^.

Thermal mass or volumetric heat capacity which is defined as $m= \rho . c$ is the ability to store thermal energy.

Considering the density of 1400 and 1650 kg/m^3^ and specific heat capacity of 930 and 880 J/kg K for fly ash and burnt clay bricks respectively, the various steady state parameters for both the bricks are calculated and shown in Table 6.

**Table 6:** Steady state parameters for fly ash and burnt clay bricks^30,31^.

Parameters	*m* (× 10^6^) (J m^−3^ K^−1^)	U-value (W/m^2^ K)	$\alpha$(× 10^−7^) (m^2^/s)	Thermal resistance (m^2^ K/W)	$\xi$(J m^−2^ K^−1^ s^0.5^)	$\lambda$(W/m K)
Fly ash bricks	1.3	0.5–1.2	3.0	2	739	0.4–0.5
Burnt clay bricks	3	2–3	5.5	0.5	1067	0.8

**Figure 8:**
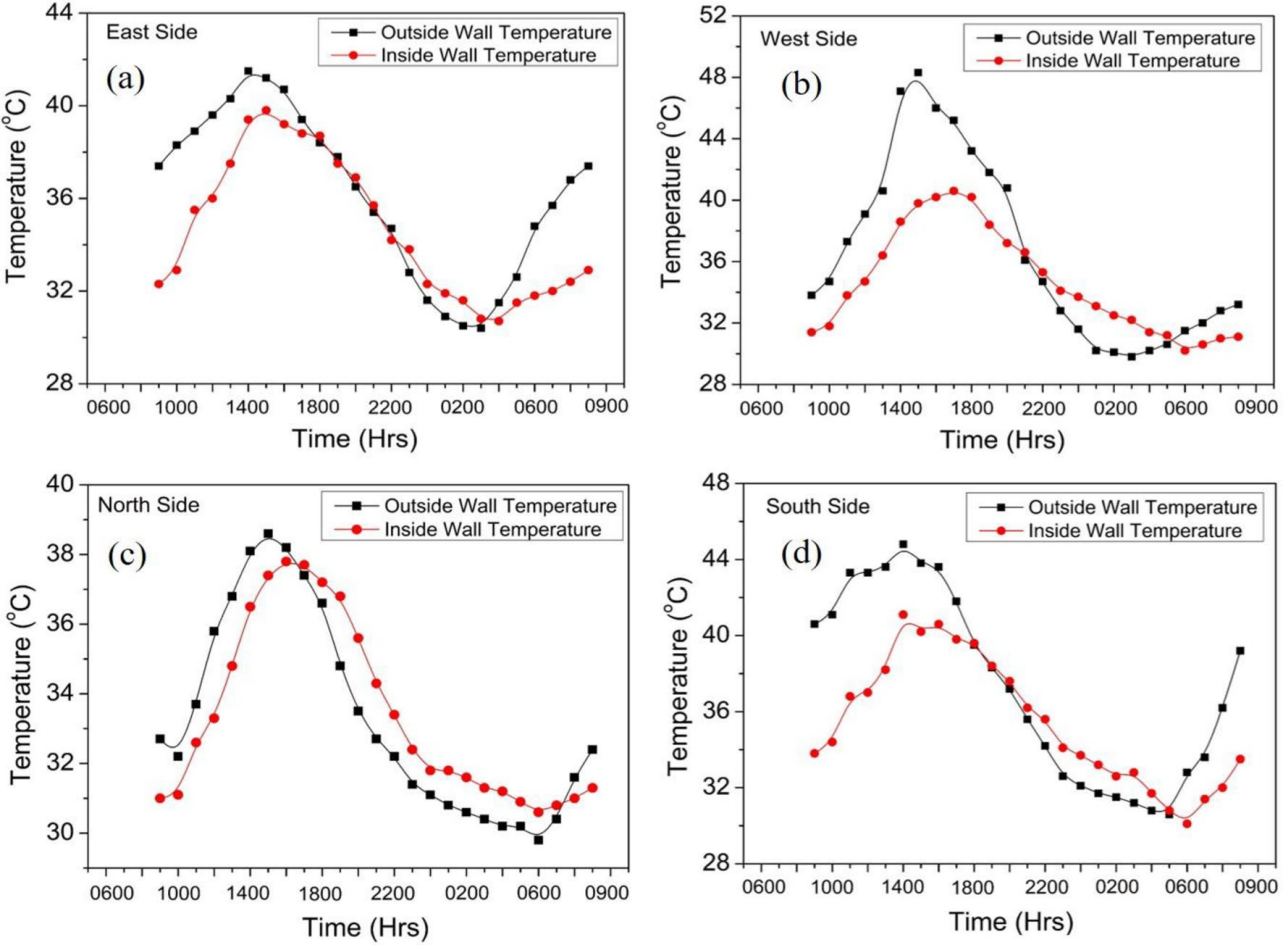
Wall temperature variation of agro-forestry waste based fly ash brick wall envelope.

**Figure 9:**
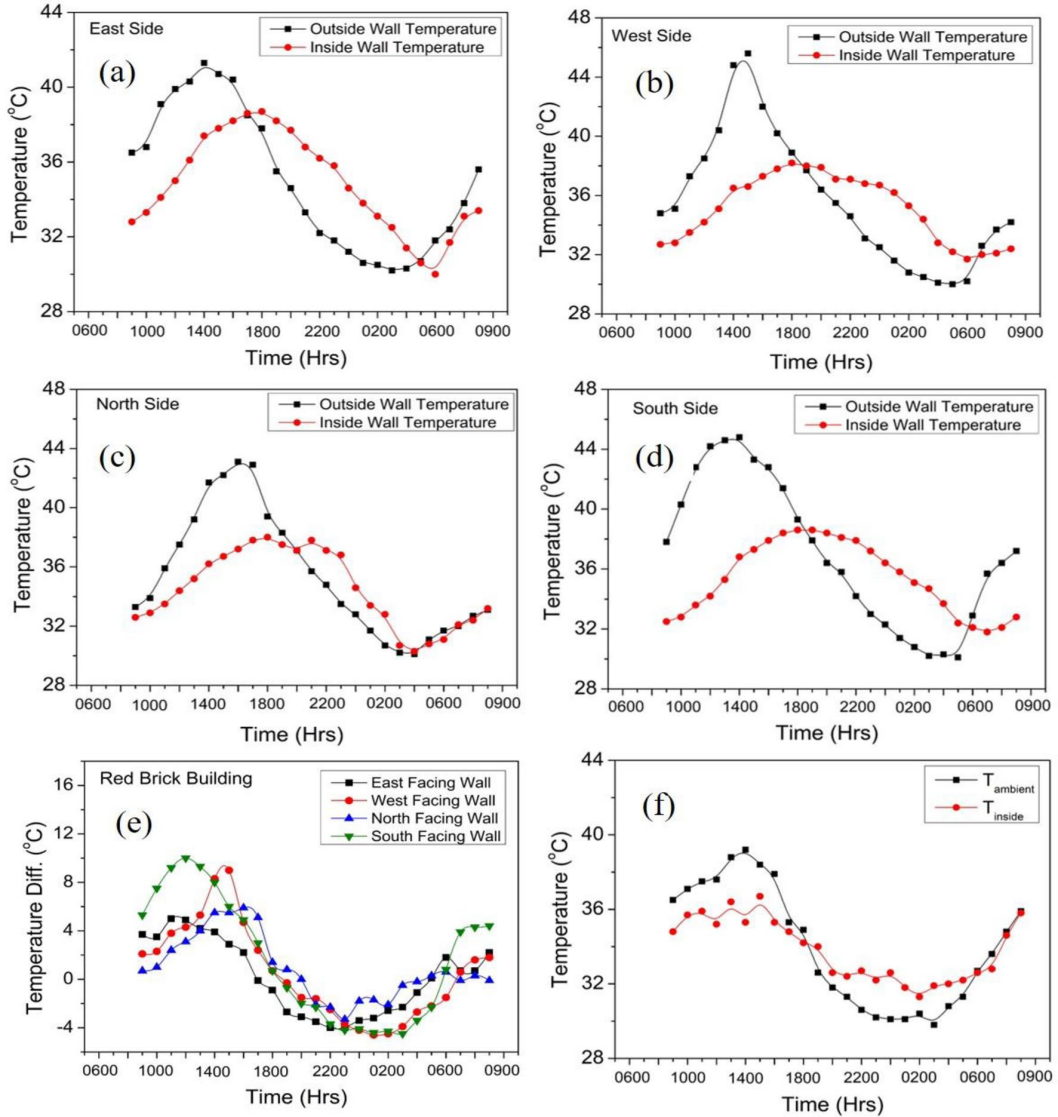
Wall temperature variation of burnt clay brick wall envelope.

As evident from the Table 6, the fly ash bricks have lower thermal mass ($m$), lower U-value, thermal diffusivity ($\alpha$) and effusivity ($\xi$) as compared with burnt clay bricks revealing better thermal insulation and low thermal energy storage capacity. Walls with lower U-value shows a better performance to indoor surface temperature variations. Figure 8 shows outer and inner wall temperature variations for different directions of the fly ash brick building. The maximum observed temperature for outer surface walls varies over the range of 38–48 °C and minimum temperature of 28–30 °C. Whereas, the inner maximum wall temperature varies over the range of 36–40 °C and minimum temperature of 29–32 °C, recorded for 24 h period. The higher inner wall temperature is attributed due to metallic sheet roof and double glazed window on west facing wall which causes the entrapment of heat energy due to non-ventilated condition of the building. The outer and inner wall temperature variations are shown in Fig. 9 for different directions of the burn clay brick building. The maximum observed temperature for outer surface walls varies over the range of 40–46 °C and minimum temperature of 29–30 °C. Whereas, the inner maximum wall temperature varies from 36 to 38 °C and minimum temperature between 29 and 31 °C, observed for 24 h. The lower inner wall temperature fluctuations are attributed to higher thermal mass and subsequently higher thermal admittance of the burnt clay bricks over fly ash bricks described in next section.

#### Dynamic state thermal analysis

Non-steady state i.e., dynamic calculations allow the thermal performance of the buildings in real time condition. The temperature variations of the external/outside environment have a profound effect on building envelope and dynamic properties viz., time lag, decrement factor, thermal damping, and thermal admittance^30,53,54^. Thermal admittance of a material (*Y*) can be defined as ability to absorb heat energy and release it over a period of time after the removal of heat source. This is an indicator of the thermal storage capacity (thermal mass) of a building or wall material. Thermal admittance is expressed in W/(m^2^K), where the higher the admittance value, larger the thermal storage capacity of the wall material. The large value of thermal admittance (*Y*) indicates lower temperature fluctuations inside the buildings. Typical admittance values are based on a 24-h temperature cycle. It measures the heat flow rate entering the internal surface of a wall as a response to a unit cyclic temperature fluctuation. The thermal admittance of both the buildings have been calculated by the transmission matrix method.

Time lag and decrement factors are critical thermal performance characteristics that influence the heat storage capabilities of any materials. Time lag (Φ) is the time difference between the maximum temperature at the outer and inner walls with periodic heat flow through the test specimen^30,53^.

The time lag can be written as:

28 $$\Phi = {t}_{{T}_{in\left(max\right)}}- {t}_{{T}_{out\left(max\right)}}$$

where, ${t}_{{T}_{in\left(max\right)}}$ and ${t}_{{T}_{out\left(max\right)}}$ are the time of day when the inside and outside surface temperatures reach maximum value.

Another parameter i.e., decrement factor ($DF$) may be defined as:

29 $$DF= \frac{{T}_{in({\text{max}})}- {T}_{in({\text{min}})}}{{T}_{out({\text{max}})}- {T}_{out({\text{min}})}}$$

where, ${T}_{in({\text{max}})}$, ${T}_{in({\text{min}})}$ are maximum and minimum inside wall temperatures and ${T}_{out({\text{max}})}$, ${T}_{out({\text{min}})}$ are maximum and minimum outside wall temperatures.

Thermal damping is also an important characteristic based on the thermal resistance of the material (expressed in percent) which can be written as

30 $$D= \frac{{T}_{o}- {T}_{in}}{{T}_{o}}\times 100$$

where, ${T}_{o}$ and ${T}_{in}$ are maximum outside and inside wall temperatures, respectively.

For thermal admittance calculation, the transmission matrix $Z$ of the multi-layered wall is obtained through the product of the matrices related to each layer, including the transmission matrix containing the film thermal resistances:

31 $$\left[\begin{array}{cc}{z}_{1}& {z}_{2}\\ {z}_{3}& {z}_{4}\end{array}\right] = \left[\begin{array}{cc}1& {R}_{si}\\ 0& 1\end{array}\right]\prod_{1}^{n}\left[\begin{array}{cc}{z}_{1}& {z}_{2}\\ {z}_{3}& {z}_{4}\end{array}\right]\left[\begin{array}{cc}1& {R}_{so}\\ 0& 1\end{array}\right]$$

The thermal admittance function $Y$ can be written as $\frac{{Z}_{4}}{{Z}_{2}}$ (W/m^2^K), $n$ is the number of homogeneous layers, *R*_*so*_ and *R*_*si*_ are film thermal resistances^54,55^.

The elements of the transmission matrix can be written as:

32 $${z}_{1}={z}_{4}={\text{cosh}}(t+i t)$$

33 $${z}_{2 }=\frac{sinh (t+it)}{\xi (1+i)}$$

34 $${z}_{3}=\xi \left(1+i\right){\text{sinh}}(t+i t)$$

where, *i* is the imaginary part, *t* is cyclic thickness and $\xi$ is the thermal effusivity, $L$ is the wall thickness, and *P* is the time period of the cyclic energy transfer. Both cyclic thickness and thermal effusivity can be expressed as:

35 $$t= \sqrt{\frac{\pi }{P. 3600} . \frac{\rho . c}{\lambda } . {L}^{2}}$$

36 $$\xi = \sqrt{\frac{2\pi . \lambda .\rho . c}{P. 3600}}$$

$\xi$ is thermal effusivity in J m^−2^ K^−1^ s^0.5^, *ρ* is the density of the material in kg m^−3^, *c* is specific heat capacity in J Kg^−1^ K^−1^ and *P* is the number of period hours.

The outside and inside surface temperatures of fly ash and burnt clay brick building walls for all the directional faces are shown in Figs. 8 and 9 respectively. The various parameters viz., time lag, decrement factor, thermal admittance and thermal damping have been calculated and listed in Table 7.

**Table 7:** Dynamic thermal parameters of fly ash and burnt clay brick buildings.

Parameters	Time lag (h)	Decrement factor	Thermal damping (%)	Thermal admittance (W/m^2^ K)
Fly ash bricks	1	0.56	13	4.4
Burnt clay bricks	3	0.43	18	5.4

As evident from Table 7, the higher time lag, lower decrement factor and high thermal damping and large thermal admittance parameters are responsible for maintaining the indoor temperatures at nearly constant temperature for burnt clay brick building envelope. Overall, the steady state and dynamic state thermal analysis helps in understanding the performance behaviour of a building envelope and selection of materials as per the ambient weather requirement.

## Summary discussion and future studies

With the perspective of the current study, there are many possibilities and challenges regarding fly ash, agro-forestry waste and C&D waste utilisation in construction sector. The paramount challenge in agro-waste management is the lack of awareness among the farmer community towards environmental and health aspects of current and future generations. In recent times, the waste management and its utilisation in construction materials has shown a positive development which includes fly ash, C&D waste use in cement industry and construction sector. These utilisation of wastes reduces the burden on environment (excavation, processing) since most of construction materials viz., sand, aggregates and cement manufacturing relies heavily on natural resources. In the present study, an attempt to adopt the various wastes in building materials to reduce the environmental threat resulting in less carbon emission of the developed product as compared to conventional burnt clay bricks thus creating sustainable building products. According to an estimation, by replacing 1–2% of brunt clay bricks with agro-forestry waste, C&D waste based fly ash bricks can potentially reduce 0.5–1.5 million tons of CO_2_ emission. The thermal analysis of fly ash brick building has shown better thermal insulation as compared to burnt clay bricks which may provide better thermal comfort to the occupants. The future work related to the current study includes full scale fire testing of studied bricks in partition walls, sound isolation analysis and utilisation of the mentioned waste materials in additive manufacturing in construction. The practical applications of the present study involve creation of eco-friendly building products for sustainable construction practices promoting circular economy and waste to wealth creation.

## Conclusions

The present work has demonstrated the potential utilisation of fly ash, C&D waste and agro-forestry waste as green building and environmental friendly materials. The mix design of the desired materials with 3 M NaOH activation leads to mechanical strength of 3–5 MPa within density range of 1400–1600 kg/m^3^. The lower compressive strength value of fly ash bricks is attributed to low molar (3 M) presence of NaOH activator resulting in lesser dissolution of aluminosilicates which releases SiO_4_ and AlO_4_ tetrahedral units responsible of promoting polycondensation process. The thermal conductivity *‘k’* values of 0.40–0.45 W/m K, of fly ash bricks results in better thermal insulation as compared to burnt clay bricks (*‘k’*: 0.8 W/m K) which may lead to thermal comfort for building occupants^26^.

The overall CO_2_ emission for manufacturing fly ash bricks in the present study is estimated at around 14.7 gCO_2_/kg of brick as compared to 97 gCO_2_/kg of burnt clay brick. The total CO_2_ emission from construction of prototype building with fly ash bricks is ~ 43.8 kgCO_2_. The net CO_2_ emission reduction for 1000 (utilised in prototype building construction) between fly ash and burnt clay bricks is ~ 245 kgCO_2_. Agro-forestry waste, C&D waste based fly ash bricks can potentially reduce 0.5–1.5 Mt of CO_2_ emission even if 1–2% of burnt clay bricks is replaced (and restricted from manufacturing) and used in construction. The embodied energy estimation shows the agro-forestry waste based fly ash bricks is 10–15 times less energy intensive than burnt clay bricks which supports the low carbon emission values of fly ash bricks.

Thermo-physical properties both steady and dynamic state viz., U-value, thermal conductivity, thermal diffusivity, thermal mass, time lag, decrement factor and thermal admittance etc., have been evaluated. The agro-forestry waste based fly ash bricks have shown lower values of U-value, thermal conductivity, thermal diffusivity, and high thermal mass, admittance and thermal damping for burnt clay bricks which are responsible in maintaining the indoor temperature variations with minimum fluctuations. These type of bricks may be utilised in hot weather environment for thermal comfort of the building occupants with energy savings. Considering the current demand of burnt clay bricks in India, the fly ash bricks along with agro-forestry waste, C&D waste has the potential to substitute burnt clay bricks in construction which can reduce the carbon footprint, land degradation and soil nutrients arising from burnt clay bricks production which is contributing to climate change and ecological imbalance and severe human health hazards.

## Data Availability

All data generated or analysed during this study are included in this published article.

## Abbreviations

AA-FA: Alkali activated-fly ash
C&D: Construction and demolition
IS: Indian standard
ICE: Inventory of Carbon and Energy
FCBTK: Fixed chimney bull’s trench kiln
GHGs: Greenhouse gases
GGBFS: Ground granulated blast furnace slag
LCA: Life cycle analysis
LoI: Loss on ignition
NTPC: National thermal power corporation
PM: Particulate matter
SCMs: Supplementary cementitious materials
t-km: Ton-kilometer
XPS: X-ray photoelectron spectroscopy
